# Quantitative magnetic resonance imaging of meniscal pathology ex vivo

**DOI:** 10.1007/s00256-021-03808-6

**Published:** 2021-05-13

**Authors:** Won C. Bae, Anthony S. Tadros, Tim Finkenstaedt, Jiang Du, Sheronda Statum, Christine B. Chung

**Affiliations:** 1 Radiology Service, Veterans Affairs San Diego Healthcare System, MC-114, 3350 La Jolla Village Drive, San Diego, CA 92161 USA; 2 Department of Radiology, University of California, San Diego, 9427 Health Sciences Drive, La Jolla, CA 92093-0997 USA; 3 Institute of Diagnostic and Interventional Radiology, University Hospital Zurich, University of Zurich, Zurich, Switzerland

**Keywords:** Knee, Meniscus, Ultrashort echo time, Osteoarthritis, Degeneration

## Abstract

**Objective:**

To determine the ability of conventional spin echo (SE) T2 and ultrashort echo time (UTE) T2* relaxation times to characterize pathology in cadaveric meniscus samples.

**Materials and methods:**

From 10 human donors, 54 triangular (radially cut) meniscus samples were harvested. Meniscal pathology was classified as normal (*n* = 17), intrasubstance degenerated (*n* = 33), or torn (*n* = 4) using a modified arthroscopic grading system. Using a 3-T MR system, SE T2 and UTE T2* values of the menisci were determined, followed by histopathology. Effect of meniscal pathology on relaxation times and histology scores were determined, along with correlation between relaxation times and histology scores.

**Results:**

Mean ± standard deviation UTE T2* values for normal, degenerated, and torn menisci were 3.6 ± 1.3 ms, 7.4 ± 2.5 ms, and 9.8 ± 5.7 ms, respectively, being significantly higher in degenerated (*p* < 0.0001) and torn (*p* = 0.0002) menisci compared to that in normal. In contrast, the respective mean SE T2 values were 27.7 ± 9.5 ms, 25.9 ± 7.0 ms, and 35.7 ± 10.4 ms, without significant differences between groups (all *p* > 0.14). In terms of histology, we found significant group-wise differences (each *p* < 0.05) in fiber organization and inner-tip surface integrity sub-scores, as well as the total score. Finally, we found a significant weak correlation between UTE T2* and histology total score (*p* = 0.007, *R*_*s*_^2^ = 0.19), unlike the correlation between SE T2 and histology (*p* = 0.09, *R*_*s*_^2^ = 0.05).

**Conclusion:**

UTE T2* values were found to distinguish normal from both degenerated and torn menisci and correlated significantly with histopathology.

## Introduction

Meniscal injury is one of the most common intra-articular knee derangements [1]. As a well-known risk factor for the development of osteoarthritis (OA) of the knee [2–8], meniscal injury represents the most frequent cause of orthopedic surgical intervention [1, 9]. Although magnetic resonance imaging (MRI) is the established method for the diagnosis of meniscal lesions, accuracy of conventional MR techniques ranges from 70 to 90% compared to that of surgery [10, 11]. As conventional morphologic MR assessment is limited to the detection of gross meniscal tissue loss and changes in hydration [12], it often results in late-stage diagnosis. Quantitative MRI has therefore been proposed as a method for identifying early biochemical changes of the injured meniscus.

Several quantitative MR techniques have been studied in the meniscus, with T2 mapping [13, 14] representing the most established technique. Based on work in articular cartilage, T2 relaxation times have been shown to reflect meniscal collagen architecture and water content [15, 16] as well as proteoglycan status [17–19], respectively. Due to the highly organized collagen ultra-structure of the meniscus and resultant short T2 relaxation time [20], more recent studies have focused on ultrashort echo time (UTE) techniques [12, 21–23]. These pulse sequences allow the signal to be detected much earlier after proton excitation and thereby improve quantification of MR properties. Several ex vivo studies recently have correlated conventional T2 [24] or UTE T2* [25] values of the meniscus against histologic measures, and we aim to build upon the past studies by also considering pathologic classification [26] of the menisci, often used in clinical settings.

The purpose of this study was to further elucidate the ability of quantitative MRI to characterize meniscal pathology in ex vivo specimens. We sought to investigate T2 and UTE-T2* measures of meniscal integrity using a modified arthroscopic classification as well as histopathologic grading as reference standards in cadaveric donor tissues.

## Materials and methods

### Samples

**Fig. 1:**
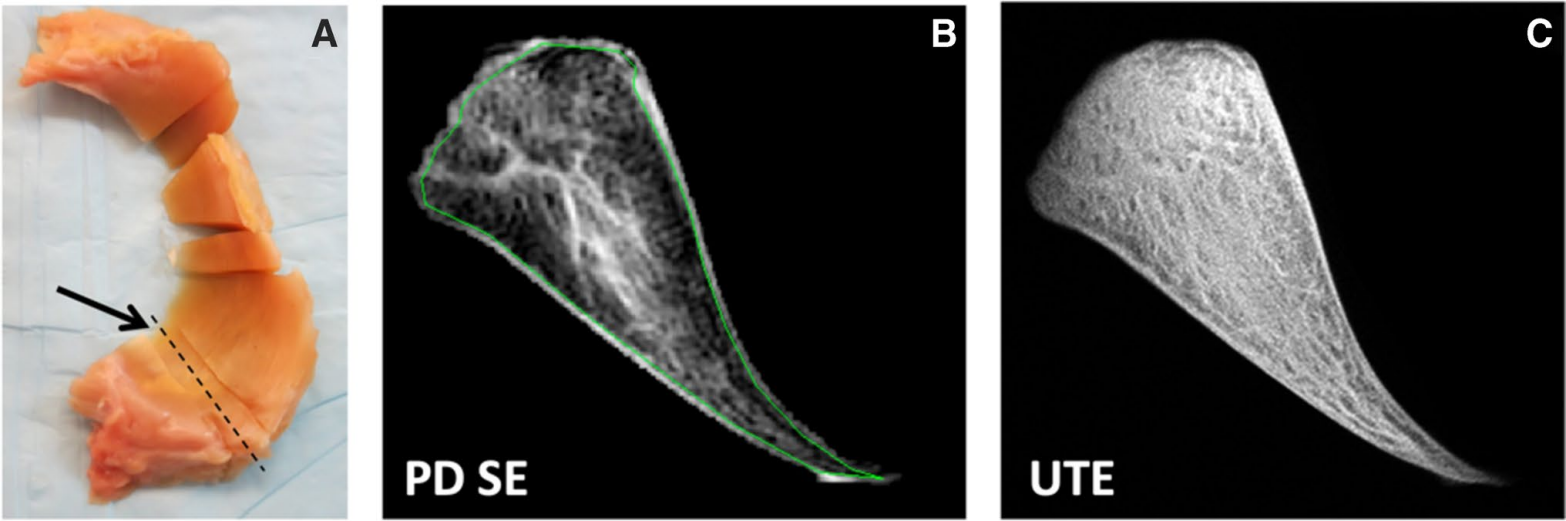
An 80-year-old male cadaveric donor with degenerated medial meniscus. **A** Cross section of the posterior meniscus (arrow) with dashed line indicating plane of MR imaging. **B** Proton density–weighted spin echo image (TE = 15 ms) with abnormal intra-meniscal signal that was graded as having mild intrasubstance degeneration. **C** Ultrashort echo time image (TE = 0.01 ms) reveals detailed fibrous architecture not seen in the conventional spin echo image

This cadaveric study was exempt from the institutional review board approval. Cadaveric knees were obtained from 10 human donors (South Texas Blood and Tissue Center, San Antonio, TX). There were 5 males, 3 females, and 2 unknown sexes. Age range was 60 to 87 years with a mean age of 79 years. Medial (*n* = 10) and lateral menisci (*n* = 8) were harvested from intact donor knee joints. Triangular pieces (*n* = 54), 5-mm thick, were harvested from the anterior horn, body, and posterior horn of each meniscus (Fig. 1A). Samples were then frozen at − 40 C° (Bio-Freezer; Forma Scientific) until imaging. Prior to imaging, samples were thawed for 3 h at room temperature and submerged in perfluorocarbon (SynQuest Labs, Alachua, FL) to reduce MR susceptibility artifacts during imaging.

### MR imaging

MR imaging was performed on a 3 T GE Signa HDX system (GE Healthcare, Milwaukee, WI) in a 1″ diameter solenoid coil. Samples were mounted in an MR-compatible device with meniscal circumferential fibers oriented parallel to B_0_. Scanning was performed in an axial plane (perpendicular to B_0_).

For morphologic examination, imaging protocol consisted of a proton density–weighted non-fat-suppressed turbo-spin echo sequence (Fig. 1B) (TR = 2500 ms, TE = 15 ms, field of view = 4 cm, matrix = 320 × 320, slice thickness = 1 mm, number of slices = 1, number of excitations = 2, flip angle = 90 degrees, bandwidth =  ± 19 kHz) and 2D UTE fat-suppressed (Fig. 1C) and non -fat-suppressed sequences (TR = 300 ms, TE = 0.01, 12 ms, field of view = 4 cm, matrix = 512 × 455, slice thickness = 2 mm, number of excitations = 2, flip angle = 45 degrees, bandwidth =  ± 25 kHz).

**Fig. 2:**
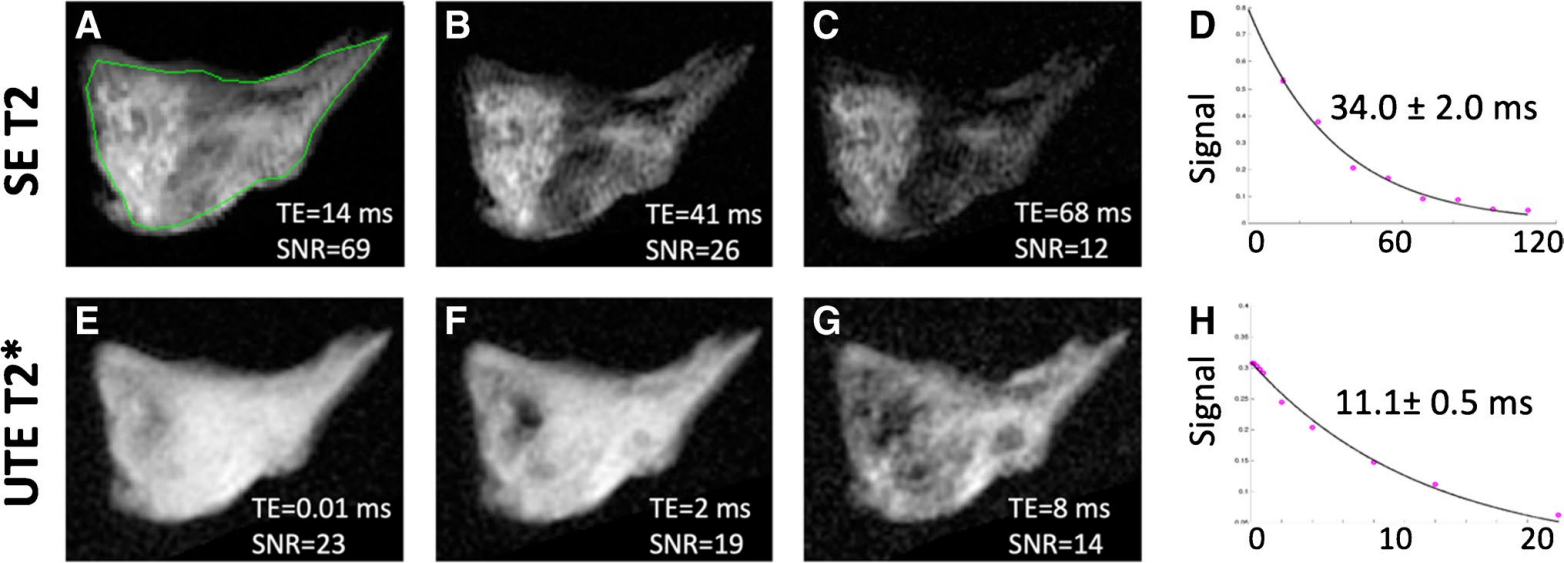
Quantitative MR data acquisition from a degenerated meniscal specimen. T2 (**A**, **B, C**) and UTE T2* (**E**, **F**, **G**) images of the meniscus obtained at increasing echo times (left to right) demonstrate corresponding decreasing signal-to-noise ratios (SNR), determined from the average intensity of the whole sample divided by background signal intensity. SE T2 (**D**) and UTE T2* (**H**) signal decay curves were subsequently generated for each set of images, from which fitted relaxation values ± standard errors were obtained. Green line (**A**) illustrates region of interest used to determine the relaxation times

Quantitative imaging protocol consisted of a 2D spin echo multi echo T2 mapping (SE T2) sequence based on Carr-Purcell-Meiboom-Gill acquisition (Fig. 2A to C) (TR = 2000 ms, TE = 14, 27, 41, 55, 68, 82, 96, 109 ms, field of view = 5 cm, matrix = 320 × 256, slice thickness = 2 mm, number of slices = 1, number of excitations = 1, flip angle = 90 degrees, bandwidth =  ± 21 kHz) and a 2D UTE T2* mapping sequence (Fig. 2E to G) (radial acquisition; TR = 100 ms, TE = 0.01, 0.1, 0.2, 0.4, 0.6, 0.8, 2, 4, 8, 12, and 20 ms, field of view = 5 cm, matrix = 256 × 255, slice thickness = 2 mm, number of excitations = 2, flip angle = 35 degrees, bandwidth =  ± 31 kHz).

### MR image analysis

**Fig. 3:**
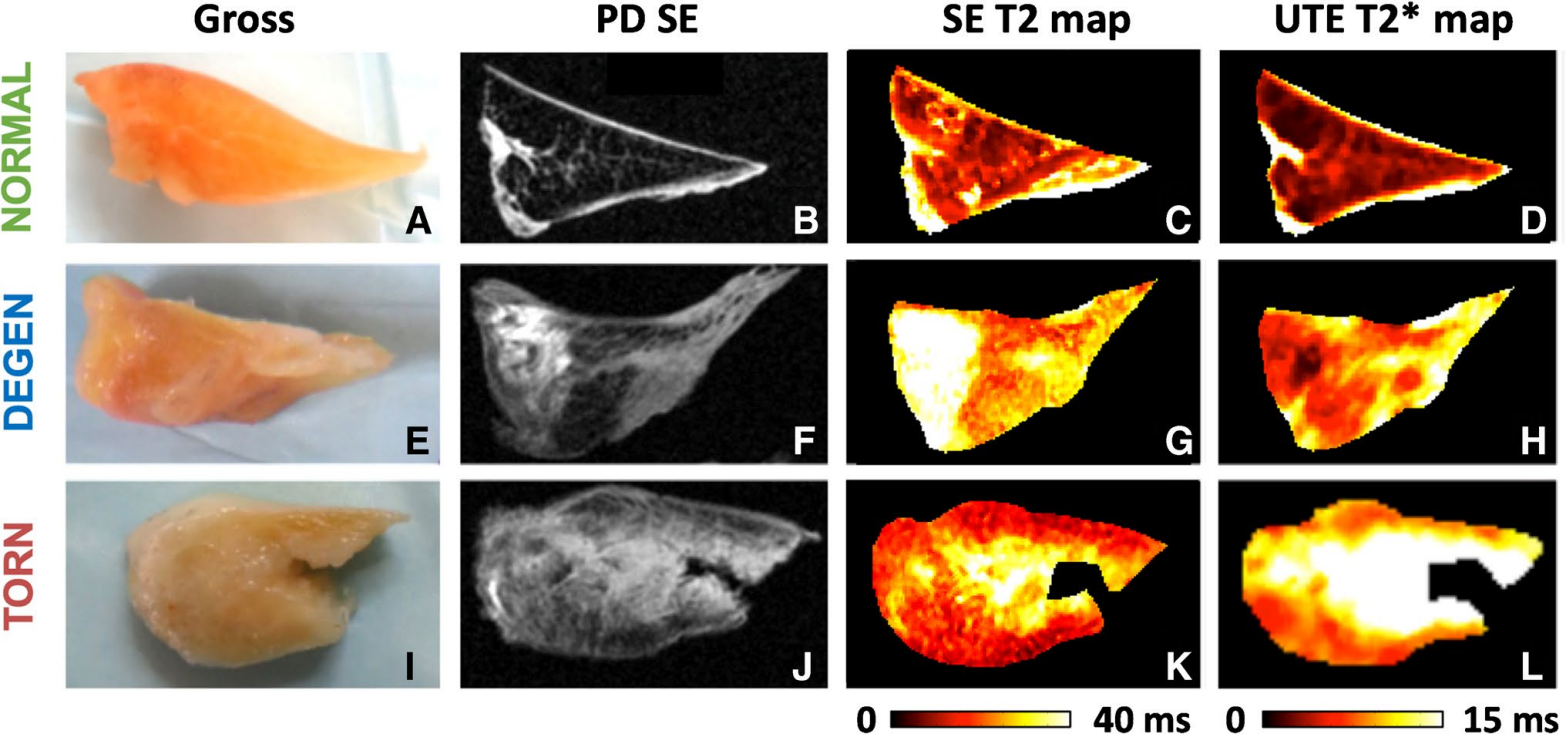
Quantitative MR data displayed for representative normal (**A**, **B**, **C**, **D**), degenerated (**E**, **F**, **G**, **H**), and torn (**I, J**, **K**, **L**) menisci. Pixel maps for SE T2 (**C**, **G**, **K**) and UTE T2* (**D**, **H**, **L**) quantitative MR data demonstrate heterogeneously increased relaxation times in degenerated and torn menisci; color bar indicates relative magnitude of T2 and T2* values. Note increased quantitative values in the torn specimen predominantly surround the tear. Gross specimen photos (**A**, **E**, **I**) and proton density–weighted spin echo (PD SE) images (**B**, **F**, **J**) demonstrate corresponding loss of tissue architecture and abnormal increased intra-meniscal signal, respectively

Regions of interest (ROIs) were manually drawn for the entire meniscus sample by one individual (AST) with 4 years of experience (Fig. 2A). In cases of meniscal tear, only meniscal tissue was included in the ROI. Mean and standard error of the mean (SEM) relaxation times for SE T2 and UTE T2* were obtained using nonlinear least square mono-exponential curve fitting (without offset) of average signal intensities (Fig. 2D and H). Color maps (Fig. 3) were also generated to compare with the average ROI values and to demonstrate spatial variations in MR values within the sample. Analysis was performed using an in-house software developed with MATLAB (MathWorks, Natick, MA).

### Gross and morphologic MR classification

Meniscus samples were classified as normal, intrasubstance degeneration (degenerated), or torn using a modified arthroscopic grading system [26] consisting of gross tissue inspection and palpation. Loss of tissue architecture and decreased firmness with probing were considered arthroscopic signs of meniscal degeneration. Surface irregularity or surface defect with extension into the meniscal substance were consistent with meniscal tear. High-resolution proton density–weighted spin echo and UTE morphologic MRI were examined. Abnormal altered MR signal within the meniscus was considered the primary indicator of intrasubstance degeneration. Linear signal extending to a meniscal surface defined meniscal tear. High-resolution conventional imaging facilitated detection of tears and abnormal signal intensity, while UTE imaging enabled visualization of detailed fibrous architecture. The classification was performed by a fellowship trained musculoskeletal radiologist with 4 years of experience who was blinded to quantitative MR data and histology.

### Histopathology

Meniscus samples were processed for histology by fixation in Z-fix (Anatech, Battle Creek, MI) for 3 days, dehydration in graded ethanol, and embedded in paraffin. Sections (5-um thick) were stained with hematoxylin and eosin (H&E) [27] to evaluate cellularity, cellular morphology, and tissue organization. Safranin-O Fast Green (Saf-O) [27, 28] staining (Figs. 5A and 6) was performed to determine the distribution of proteoglycan content.

Meniscus histopathology was based on a published grading system (Table 1) [29], evaluating surface integrity (femoral side, tibial side, inner rim; each scored from 0 to 3), cellularity (0 to 3), collagen fiber organization (0 to 3), and Safranin-O staining (0 to 3). These individual sub-scores were added to determine the total score; a score of 0 would represent a healthy normal meniscus, while the maximum score of 18 would represent a severely degenerated or abnormal meniscus. We selected standard magnification and views for each grading criteria, to ensure consistent and repeatable measurement. Two readers (TF and NA) independently performed the reading and the scores were averaged.

**Table 1:** Histopathology grading scheme

	< ––Normal Score Abnormal–– >			
Grading feature	0	1	2	3
Femoral surface irregularity	Smooth	Slight fibrillation	Moderate	Severe
Tibial surface irregularity	Smooth	Slight fibrillation	Moderate	Severe
Inner rim surface irregularity	Smooth	Slight fibrillation	Moderate	Severe
Cellularity	Normal	Diffuse hypercellular	Diffuse hypo/acellular	Hypocellular
Matrix/fiber organization	Organized; homogeneous	Organized; foci of hyaline or mucoid degeneration	Unorganized; bands of hyaline/mucoid degen.; fraying	Unorganized; fibrocartilaginous separation; severe fraying
Saf-O/fast green intensity	None	Mild	Moderate	Strong

### Statistical analysis

Descriptive statistics on cadaveric donors are reported. Effect of region (anterior, body, posterior) on the observed frequency of different meniscal pathology was evaluated using chi-square test. To determine the effects of meniscal pathology on quantitative MR properties, analysis of variance (ANOVA) was performed to compare mean relaxation times of the normal, degenerated, and torn samples. Normality of MR data was first checked with Shapiro–Wilk test. Since both SE T2 (Shapiro–Wilk *p* = 0.07) and UTE T2* (Shaprio-Wilk *p* = 0.001) data were somewhat skewed in distribution, the data were log-transformed prior to performing ANOVA. Tukey’s test was used for subsequent pairwise comparisons. Power analysis was performed using the G*Power software [30].

To determine effects of meniscal pathology on histology scores, Kruskal–Wallis test [31] was used, along with pairwise comparisons using Bonferroni adjustment of significance. Lastly, to examine the correlation between histopathology scores and relaxation times, we performed Spearman rank correlation. Correlation was performed for the individual scores (e.g., fiber organization) as well as the summed total score. Differences were considered significant at *p* < 0.05. Shapiro–Wilk test, ANOVA, Kruskal–Wallis test, and Spearman correlation were performed using the Systat software package (version 12; Systat Software Inc., San Jose, CA).

## Results

Gross specimen examination and morphologic MR grading diagnosed 17 (31% of 54 samples) meniscus samples as normal, 33 (61%) intrasubstance degeneration (degenerated), and 4 (7%) torn, all extending to the meniscal surface. The distribution of pathology by region of meniscus is presented in Table 2. While lateral and medial regions had a similar frequency of normal and degenerated menisci (chi-square *p* = 0.24), compared to anterior regions, both the body (*p* = 0.0004) and posterior (*p* = 0.0008) regions had a greater frequency of degenerated samples.

**Table 2:** Distribution of meniscal pathology by anatomic region

	Classification of pathology		
Meniscal region	Normal	Degenerated	Torn
Lateral-anterior	5	3	0
Lateral-body	1	6	1
Lateral-posterior	3	4	1
Medial-anterior	7	3	0
Medial-body	1	7	2
Medial-posterior	0	10	0
Sub total	17	33	4

**Fig. 4:**
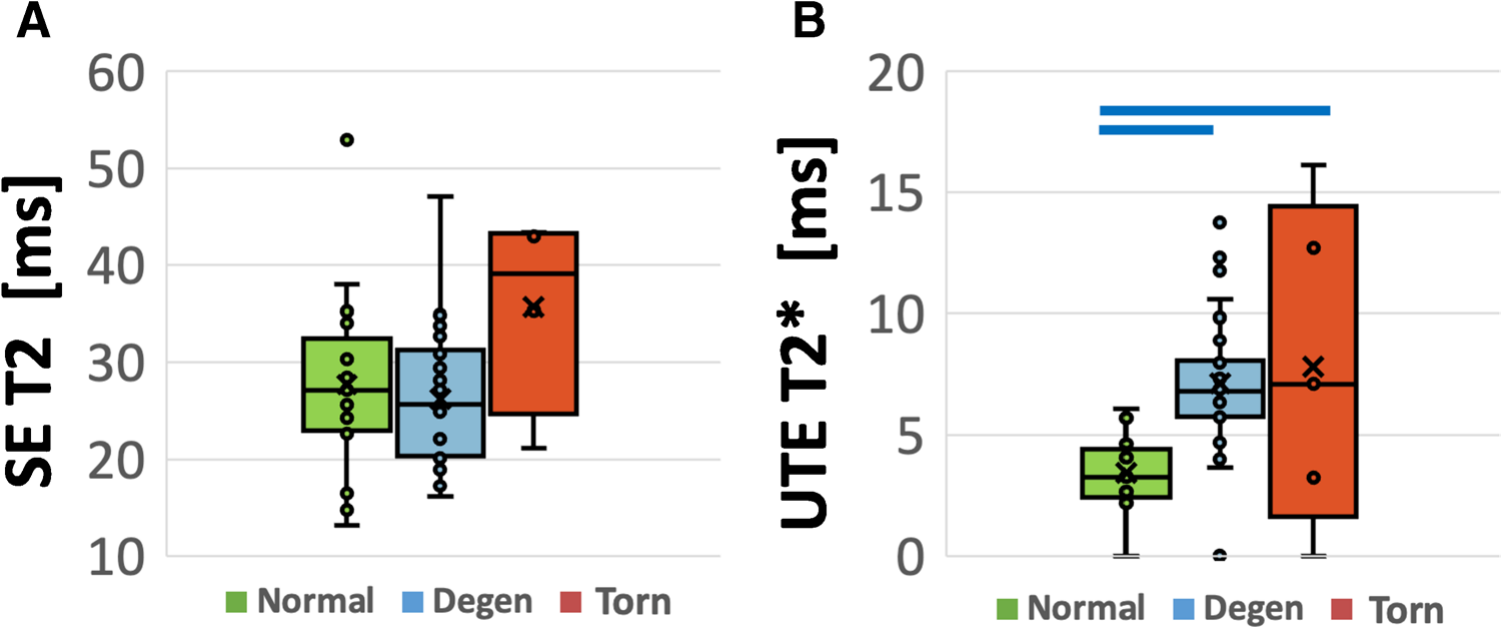
Bot plots of SE T2 (**A**) and UTE T2* (**B**) relaxation times in each experimental group, indicating data points (o), 1st to 3rd quartile (box), the minimum to maximum value (vertical bar), the median value (horizontal line within the box), and the mean value (x). While no significant differences were observed between groups (all *p* > 0.14) for SE T2 values (**A**), significant differences between normal and degenerated (*p* < 0.0001), and normal vs. torn (*p* < 0.0002) samples were found for the UTE T2* values (**B**). Blue horizontal bars on top of the plots indicate significant differences with *p* < 0.0002

Mean and standard deviation of SE T2 values (Fig. 4A) for normal, degenerated, and torn menisci were 27.7 ± 9.5 ms, 25.9 ± 7.0 ms, and 35.7 ± 10.4 ms, respectively. Mean UTE T2* values (Fig. 4B) of normal, degenerated, and torn menisci were 3.6 ± 1.3 ms, 7.4 ± 2.5 ms, and 9.8 ± 5.7 ms, respectively. Using ANOVA, UTE T2* mean relaxation times showed overall significant differences between the groups (ANOVA *p* < 0.0001; power = 0.99), as well as pairwise differences between normal and degenerated menisci (*p* < 0.0001) and between normal and torn menisci (*p* = 0.0002). In contrast, no significant differences in mean SE T2 values were observed between groups (*p* = 0.17; power = 0.33). Regional variation (in pooled samples regardless of pathology) showed no significant difference in SE T2 (*p* = 0.5) or UTE T2* values (*p* = 0.08). Two-way ANOVA could not be performed due to incomplete distribution of pathology in the three anatomic regions.

**Fig. 5:**
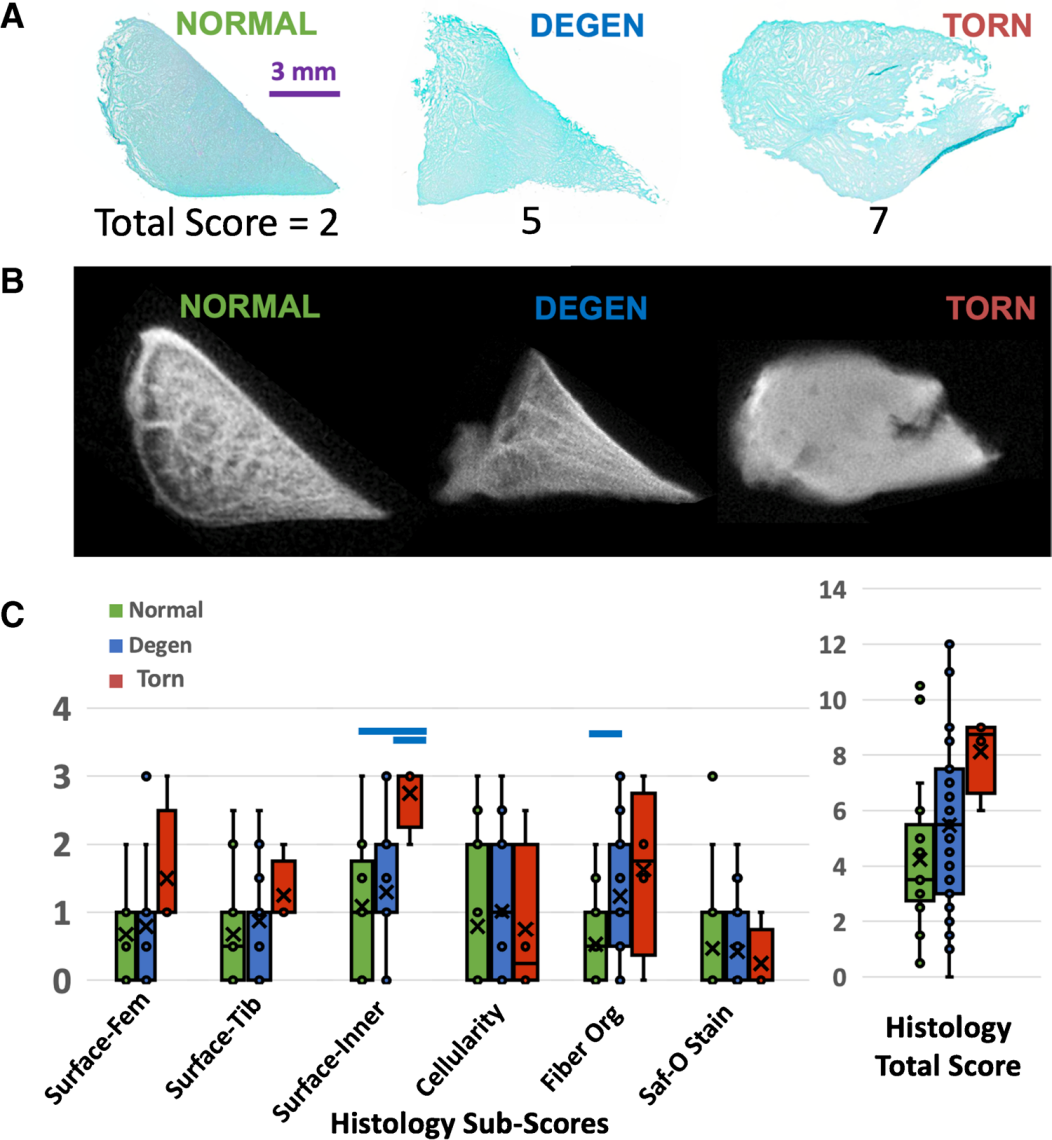
Representative Saf-O and fast green–stained histologic (**A**) and UTE MR (**B**) images of normal, degenerated, and torn samples, showing a good correspondence in gross morphology. (**C**) Box plot of histology scores show a general trend of elevated scores from normal to degenerated to torn samples, with notably increased sub-scores of inner rim surface irregularity and fiber organization, indicated by blue horizontal bars (each *p* < 0.05)

**Fig. 6:**
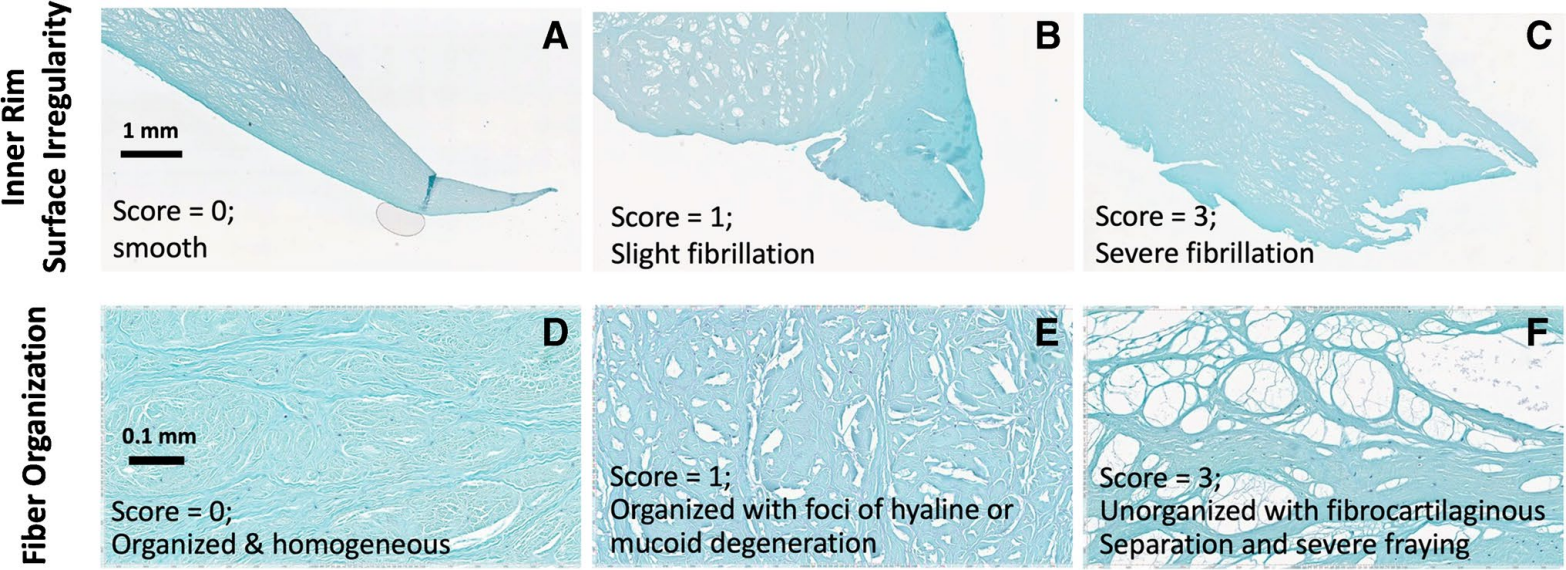
Examples of micrographs used for inner rim surface irregularity (**A**, **B**, **C**) and fiber organization (**D**, **E**, **F**) histology scoring

Histology images (Fig. 5A) showed good visual correspondence with UTE MR images (Fig. 5B) while additionally revealing micro-structural alterations. In terms of histologic grades, we found significant differences in several individual sub-scores as well as in the total score between groups (Fig. 5C). We found that the surface integrity of the inner rim (Fig. 6A, B, C) was significantly worse for torn samples compared to all others (each *p* < 0.05), while the fiber organization (Fig. 6D, E, F) of degenerated samples were worse (*p* < 0.01) compared to the normal samples. However, there was no statistically significant difference between the groups in the histology total score (all *p* > 0.08).

**Fig. 7:**
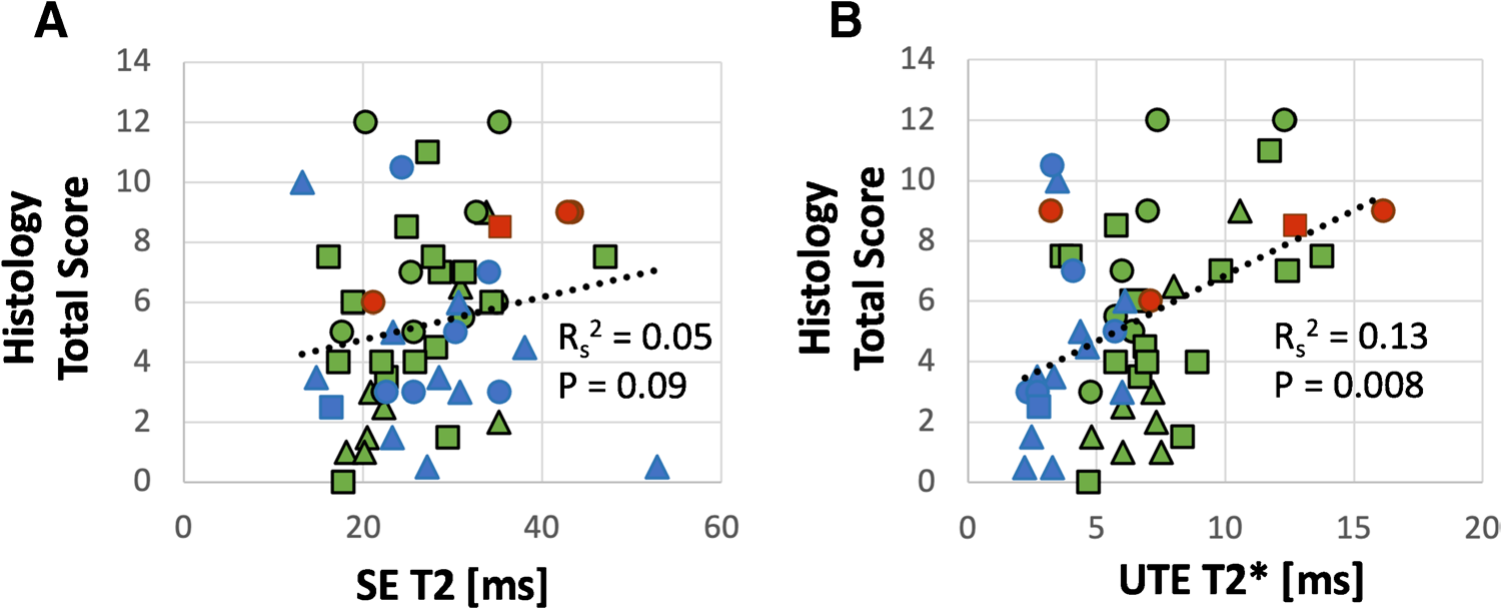
Correlation between histology total score and SE T2 (**A**) or UTE T2* (**B**) relaxation times. Symbols indicate the anatomic region where the sample was harvested: triangle = anterior horn, circle = body, square = posterior horn. Colors indicate meniscal pathology: green = normal, blue = degenerated, and red = torn. When all data points were pooled for correlation, while there was no correlation between SE T2 and histology score (**A**), UTE T2* correlation (**B**) was positive and weak

We found no correlation between SE T2 values and histology total scores (Fig. 7A), with a *R*_*s*_^2^ = 0.05 and *p* = 0.09. In contrast, between UTE T2* and histology total scores (Fig. 7B), we found a statistically significant (*p* = 0.008) but weak positive correlation with a *R*_*s*_^2^ = 0.13. When correlated vs. histology sub-scores, we found a significant (*p* = 0.002), weak, and positive correlation (*R*_*s*_^2^ = 0.18) between UTE T2* and fiber organization, and a nearly significant weak positive correlation vs. surface integrity of the inner rim (*p* = 0.054, *R*_*s*_^2^ = 0.07). All other correlations with sub-scores were not statistically significant.

## Discussion

In this study, quantitative MRI using SE T2 and UTE T2* techniques was used to evaluate cadaveric human menisci with modified arthroscopic classification of pathology and compared against histology. Spin echo T2 (SE T2) relaxation times of the meniscus were unable to distinguish normal from (intrasubstance) degenerated or torn menisci. These results conflict with a previous work by Rauscher et al., which demonstrated increased meniscal T2 values in patients with clinically mild or severe OA compared to that in healthy controls [13]. Similar findings were shown in a subsequent study that found elevated T2 values in patients with meniscal tears compared to patients without tears, as determined by morphologic MRI [14]. We suspect that our discrepant results are attributable to several confounding factors. First, our tissue donors were of old age (average 79 years), which could have led to high sample variability and overall loss of tissue integrity even in relatively normal samples, reducing the difference in SE T2 values between the experimental groups. Secondly, our degenerated samples often had focal intrasubstance degeneration, but our analysis was performed using a global region of interest. This may have reduced the sensitivity of T2 measurement to the focal pathology. Thirdly, it is possible that our samples had changes in short T2 components that were inadequately quantified using the Carr-Purcell-Meiboom-Gill (CPMG) technique. This sequence uses a relatively long minimum TE (14 ms in this study) and long TE spacing (> 10 ms), which can result in suboptimal sampling of the T2 decay curve. Other T2 quantification techniques, such as gradient echo with magnetization preparation [32] and double-echo steady-state [33] techniques, may be more sensitive to changes in short T2 components. These sequences take advantage of a shorter minimum TE (2 to 4 ms) and shorter TE spacing. In particular, both of the aforementioned studies [13, 14] that correlated T2 values with meniscal injury employed SPGR sequences combined with nonselective T2 preparation. Despite all of these observations, when meniscal degeneration without tearing has been compared to normal menisci, a previous work has been unsuccessful in differentiating these entities using T2 mapping [14]. Meniscal quantification using UTE T2* technique has therefore been an area of active interest.

In contrast to SE T2, our UTE T2* relaxation times were able to distinguish normal from both degenerated and torn menisci. These results are consistent with previous studies, which found UTE T2* mapping capable of detecting subclinical meniscal degeneration in ACL-injured patients without meniscal tears compared to that in uninjured subjects [12, 22]. Given the complex collagen matrix of the meniscus, it is composed of approximately half bound and half free water pools that result in multicomponent transverse relaxation of magnetization [34]. UTE T2*, as calculated from a mono-exponential fit in this study, therefore represented a combination of both short and long T2* decay components. Histologic analyses have showed that osteoarthritic menisci are characterized by collagen loss and disorganization [35]. Consequently, changes related to collagen breakdown and its bound water fraction were likely responsible for increased UTE T2* relaxation times in degenerated and torn specimens. This premise is supported by a recent analysis by Juras et al., which showed higher sensitivity and specificity for meniscal lesions using the short component of T2* compared to the mono-exponentially calculated T2* [36]. Our UTE T2* results also stress the limitation of our CPMG protocol, which likely only effectively evaluated longer T2 components and therefore was insensitive to structural alterations in the meniscus.

Histology results further supported higher sensitivity of UTE T2* technique to meniscal pathology compared to SE T2. We found that, while the histology sub-scores generally worsened from normal-to-degenerated-to-torn menisci, the most marked changes were in the collagen fiber organization (Fig. 5C). Fiber organization was normal and homogeneous (Fig. 6D) in majority of the normal samples but became more disorganized with foci or bands of hyaline or mucoid degeneration (Fig. 6E), along with fraying (Fig. 6F), in degenerated and torn samples. These alterations corresponded with marked increases in UTE T2* values. These results are in line with a past study by Nebelung et al. [25] who also found markedly increased UTE T2* values in menisci with high histologic grades in samples retrieved from total knee surgery.

Potential clinical applications of quantitative MRI of the meniscus have focused on the post-operative knee. For example, Chu et al. demonstrated normalization of elevated UTE T2* values in intact menisci two years following ACL reconstruction [22]. The relationship of UTE T2* and meniscal healing was further investigated in a preliminary study evaluating patients 6-months and 12-months post-meniscal surgery, although no correlation with morphologic MRI was found [23]. Additional work is needed to determine whether these measurements can possibly serve as biomarkers for treatment efficacy and help guide the course of post-operative patients. While the results of this demonstrated that lower UTE T2* values (~ less than 5 ms) were generally indicative of healthier menisci, it is premature to guide clinical decisions, without further examination of how the T2* values change with acquisition resolution and different imaging planes affected by the magic angle effect.

There are several limitations to this study. First, our study specimens were obtained from cadaveric donors. While this may have affected tissue hydration, it would be expected to have a negligible effect on comparative results as it would impact all specimens. Samples were previously frozen, which is known to impact tissue microstructure through enlargement of collagen fibrils [37], though its effect on quantitative MR properties is uncertain. Further specimen preparation using a perfluorocarbon submersion may have also influenced tissue signal; however, this suspension has been shown to have no significant effect on T2 and T2* values [38]. Second, ROIs of the entire meniscus sample were measured, when it is known that meniscal biochemical changes and quantitative MR relaxation times vary by meniscal zone [35, 39–41] or occur focally. Although not used in this study, pixel maps of MR properties would be useful for detecting zonal or focal alterations. Considering the zonal/focal variation could provide additional sensitivity for quantitative analyses, as pathologic changes may initiate focally then spread to adjacent regions. Third, there were very few torn menisci found in our pool of samples, which limited our assessment for regional differences in this group. Lastly, in order to minimize magic angle effects, menisci were non-physiologically oriented with circumferential fibers parallel to B_0_. As a result, absolute relaxation times are not generalizable. However, all specimens underwent a constant experimental procedure, and therefore, the relationships between measured values are expected to remain valid.

In conclusion, UTE T2* values were found to distinguish normal from both degenerated and torn menisci and correlated significantly with histopathology. This noninvasive technique may provide an advantage over arthroscopy, which is limited to the examination of meniscal surface areas. Detection of intrasubstance meniscal abnormality may ultimately allow for improved monitoring of disease as well as earlier non-operative treatment.
